# Full-length 16S rRNA gene sequencing by PacBio improves taxonomic resolution in human microbiome samples

**DOI:** 10.1186/s12864-024-10213-5

**Published:** 2024-03-25

**Authors:** Elena Buetas, Marta Jordán-López, Andrés López-Roldán, Giuseppe D’Auria, Llucia Martínez-Priego, Griselda De Marco, Miguel Carda-Diéguez, Alex Mira

**Affiliations:** 1 Genomics & Health Department, FISABIO Foundation, Valencia, Spain; 2 Department of Periodontics, Faculty of Medicine and Dentistry, University of Valencia, Valencia, Spain; 3 Sequencing and Bioinformatics Service, Fundació Per Al Foment de La Investigació Sanitària I Biomèdica de La Comunitat Valenciana (FISABIO-Salut Pública), València, Spain; 4 CIBER Center for Epidemiology and Public Health, Madrid, Spain

**Keywords:** 16S rRNA gene, PacBio, Illumina, Taxonomy, Sequencing, Microbiome

## Abstract

**Background:**

Sequencing variable regions of the 16S rRNA gene (≃300 bp) with Illumina technology is commonly used to study the composition of human microbiota. Unfortunately, short reads are unable to differentiate between highly similar species. Considering that species from the same genus can be associated with health or disease it is important to identify them at the lowest possible taxonomic rank. Third-generation sequencing platforms such as PacBio SMRT, increase read lengths allowing to sequence the whole gene with the maximum taxonomic resolution. Despite its potential, full length 16S rRNA gene sequencing is not widely used yet. The aim of the current study was to compare the sequencing output and taxonomic annotation performance of the two approaches (Illumina short read sequencing and PacBio long read sequencing of 16S rRNA gene) in different human microbiome samples. DNA from saliva, oral biofilms (subgingival plaque) and faeces of 9 volunteers was isolated. Regions V3-V4 and V1-V9 were amplified and sequenced by Illumina Miseq and by PacBio Sequel II sequencers, respectively.

**Results:**

With both platforms, a similar percentage of reads was assigned to the genus level (94.79% and 95.06% respectively) but with PacBio a higher proportion of reads were further assigned to the species level (55.23% vs 74.14%). Regarding overall bacterial composition, samples clustered by niche and not by sequencing platform. In addition, all genera with > 0.1% abundance were detected in both platforms for all types of samples. Although some genera such as *Streptococcus* tended to be observed at higher frequency in PacBio than in Illumina (20.14% vs 14.12% in saliva, 10.63% vs 6.59% in subgingival plaque biofilm samples) none of the differences were statistically significant when correcting for multiple testing.

**Conclusions:**

The results presented in the current manuscript suggest that samples sequenced using Illumina and PacBio are mostly comparable. Considering that PacBio reads were assigned at the species level with higher accuracy than Illumina, our data support the use of PacBio technology for future microbiome studies, although a higher cost is currently required to obtain an equivalent number of reads per sample.

**Supplementary Information:**

The online version contains supplementary material available at 10.1186/s12864-024-10213-5.

## Background

The development of sequencing platforms has revolutionized the study of microbial communities. In order to study the taxonomic composition of a bacterial community, the sequencing of the 16S rRNA gene has been the gold standard [1, 2]. This gene is around 1,500 bp long and has 9 variable regions that collect the main evolutionary changes among microbial taxa [3]. Since the nineties, the use of the whole 16S rRNA gene has been the most accepted approach in microbial taxonomic annotation, which could only be reached through first generation Sanger sequencing. This technology became obsolete with the advent of second-generation sequencers, such as pyrosequencing or Illumina sequencing (based on fluorescently labeled reversible terminator), which significantly increased the number of reads obtained [4, 5]. Consequently, the understanding of the role that these communities play in nature and in the human body was considerably improved. The so-called meta-barcoding approach is based on amplification of the 16S rRNA gene through PCR using universal primers targeting one or several adjacent variable regions of this gene, followed by high-throughput sequencing of the obtained amplicons. This approach has several drawbacks. Firstly, the selection of the primers for the amplification influences the diversity due to the differences in coverage and phylum spectrum achieved [6]. In addition, it is known that after approximately 10 cycles amplifying a mixed template, the PCR product ratios can be significantly biased towards over-amplifying some bacterial groups [7]. This is not a problem when shotgun, metagenomic sequencing is used. However, in the case of host-associated microbiome analysis, samples such as tissues or biopsies usually have an elevated concentration of host DNA which requires a high sequencing depth to obtain sufficient coverage of the microbial genomes [8]. The need for high sequencing depth therefore increases the cost and has prevented the extended use of shotgun methodologies. Secondly, the length of Illumina reads (max. 2 × 300 bp) limits the taxonomic annotation power of the obtained sequences and only a genus taxonomic rank can be achieved with reliable accuracy [9]. This is particularly important for bacterial genera that contain multiple species with highly similar 16S rRNA gene sequence, for example streptococci or the *Escherichia/Shigella* group, or with different ecologies (e.g. specific virulence factors) [10, 11]. Considering that species from the same genus can be associated with disease or health it is important to assure the annotation at the lowest possible taxonomic rank, and computer simulations have shown that this is improved by the extension of the read’s length analyzed [9, 12].

The combination of full 16S rRNA gene sequencing with a high sequencing output is nowadays possible because of 3rd generation sequencers. Oxford Nanopore Technologies developed the MinION sequencer which uses single molecule sequencing that produces longer reads than 2nd generation technologies [13]. Some researchers have used this technology to characterize microbial communities although the individual reads quality was an important obstacle for its routine use in meta-communities’ description procedures [14]. Since 2009, another stakeholder into the sequencing technologies market was Pacific Biosciences (PacBio), entering the 3rd generation sequencing scenario with the Single Molecule Real-Time (SMRT) system [15].

Although these approaches had initially entered the market with high sequencing error rates, both technologies have lately improved their quality output by the use of enhanced chemistry and also through deep learning protocols [16]. The PacBio Sequel system improved significantly the sequencing quality with the development of circular consensus sequencing (CCS) protocols which generates highly accurate long high-fidelity reads, also known as HiFi reads [17]. Callahan et al., demonstrated that this technology offers a single-nucleotide resolution by the use of the widely used DADA2 approach based on Amplicon Sequence Variant (ASV) classification [18]. This and Johnson studies [12] used stool samples as proof of concept for describing complex microbiome communities.

Despite its potential, the use of full length 16S rRNA gene (FL-16S rRNA gene) sequences for human microbiome studies is still scarce. In the case of oral microbiota, only a few projects have analyzed saliva or oral biofilms (dental plaque) with the RSII platform [19–21]. In addition, early dental plaque formed on hydroxyapatite disks from young adults, the saliva and supragingival plaque of children with caries and the supragingival plaque of elderly donors have been explored using the new and improved PacBio Sequel system [22–24]. However, there is a lack of experimental evidence about how the new long reads technologies, especially SMRT PacBio, may provide biased estimates of microbial composition and to what degree do they improve taxonomic assignment resolution in comparison to 2^nd^ generation sequencers.

The performance of long-read sequencing was already evaluated for different applications such as bacterial genome assembly or viral metagenomics by others [25, 26]. Considering that metataxonomy studies based on FL-16S rRNA gene will be standardized in the near future, it is necessary to evaluate the differences in taxonomic resolution and the methodological effect on bacterial proportions due to the use of FL-16S rRNA gene instead of the short reads sequencing spanning few variable regions. As far as we know, only Zhang et al. compared the outcome of both strategies on microbiome samples from 5 healthy children comparing the FL-16S rRNA using PacBio RSII platform versus the distributions obtained by the Illumina MiSeq platform over the V3-V4 variable regions [27].

In the current manuscript we evaluate the variations in bacterial composition caused by sequencing platforms through the comparison of saliva, oral biofilm (subgingival plaque) and faecal samples sequenced by Illumina (V3-V4 hypervariable regions) and PacBio sequencing. DNA from each microbiome was amplified targeting the V3-V4 regions using universal primers as described in Klindworth et al., 2013 [6] using Illumina MiSeq platform; and the full length 16S rRNA amplified using the 27F and 1492R primers from Weisburg et al. [28], with the PacBio standard protocol. Moreover, in order to evaluate which platform represented more accurately the original communities, two mock communities were also sequenced by both methodologies.

## Materials and methods

### Participants

**Fig. 1:**
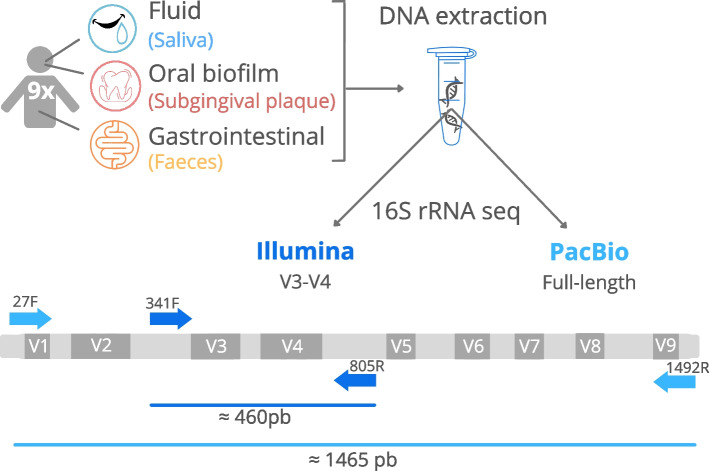
Workflow overview of the comparative analysis between Illumina and PacBio sequencing presented in the current manuscript. The variable regions of the 16S rRNA gene are represented as grey quadrangles (V1-V9). Primers used for DNA amplification in Illumina and PacBio are shown using dark and light blue arrows, respectively. The size of the amplified fragment in Illumina and PacBio is represented using a dark and light blue line, respectively

For the execution of this study, nine patients were recruited at the dental clinic at the University of Valencia (Fig. 1). All subjects were evaluated by the same dental specialist. The participants selected for the research had to meet the following inclusion criteria: periodontitis of stage III and IV grades A-B (periodontitis is an oral inflammatory disease induced by a dysbiotic biofilm, causing loss of the supporting tissues of the tooth, the gum and bone; stages III and IV are advanced phases of the disease [29]), have 20 or more teeth in the mouth, be within an age range of 30 to 70 years, be non-smokers or smoke less than 10 cigarettes a day. The applied exclusion criteria were smoking more than 10 cigarettes daily, having received antibiotic treatment in the last month or having used mouth antiseptics in the last two weeks. All patients expressed their consent to participate in the study and signed the corresponding informed consent. This research was approved by the ethics committee of the University of Valencia (registration number 1601392) and was conducted in compliance with the ethical principles established in the Declaration of Helsinki of 2008.

### Sample collection

Faecal samples were auto-collected by patients in the 24 h prior to dental visit in 5 ml of RNAlater (Invitrogen by Thermo Fisher Scientific, RNA later SoIn Cat. No AM7021) (1:1) and taken to the clinic. At the clinic, up to 2 ml of unstimulated saliva was collected and both samples were stored at 4 ºC. For the subgingival plaque biofilm samples, four paper points were placed by the odontologist into the periodontal pockets during 1 min and afterwards were kept in 1 ml of RNAlater. On the same day of collection, all sample types arrived at the laboratory and were stored at -80 ºC until processing.

### DNA isolation

Previous to DNA extraction, subgingival plaque samples were vortexed for 2 min in order to separate bacteria from the paper points. After that, paper points were removed, and the samples centrifuged for 30 min at 13,000 rpm. To obtain the bacterial pellet in saliva samples, 250 μl of saliva were centrifuged for 10 min at 13,000 rpm. In the case of faecal samples, 5 ml of phosphate-buffered saline (PBS) were added to the same amount of RNALater/faecal suspension and were mixed properly. Subsequently, it was centrifuged for 3 min at 2000 rpm and the supernatant was transferred to a fresh tube. As in saliva, 250 μl of this supernatant were centrifuged to pellet bacteria.

After centrifuged all sample types were treated with the same protocol as follows; we discarded the supernatant and resuspended the pellet in 100 μl of PBS to proceed with the lysis [10]. Then, 130 μl of lysis buffer was added to microbial suspension, in combination with 10 μl of enzymatic cocktail mix (25 mg/ml of lysozyme (Appli chem, Cat. No A4972,0001) 1.25 KU/ml of lysostaphin (Sigma-Aldrich, Cat. No SAE0091-2MG), 0.625 KU/ml of mutanolysin (Sigma-Aldrich, Cat. No SAE0092-10KU) and 125 KU/ml of zymolase (Sigma-Aldrich, Cat. No SAE0098-20KU). After 1 h at 37 ºC, 20 μl of glucanex 1% (10 min at 60 ºC) (Sigma-Aldrich, Cat. No L1412-5G) and proteinase K were added and incubated for an additional 15 min at 65 ºC followed by a boiling step (10 min, 95 ºC). Then, DNA was isolated by MagNA Pure LC 2.0 Instrument (Roche Diagnostics, Risch-Rotkreuz, Switzerland), using the MagNA Pure LC DNA Isolation Kit III for Bacteria and Fungi (Roche Diagnostics, Cat. No. 03 264 785 001) following the manufacturer’s instructions. Once the DNA was quantified using QubitTM 1X dsDNA HS Assay Kit according to the manufacturer's instructions, we split it for sequencing in the two platforms. One faecal sample contained a low amount of DNA and was therefore not included in the sequencing.

### Mock communities

Two in-house mock communities containing *Actinomyces oris, Fusobacterium nucleatum, Rothia dentocariosa, Streptococcus oralis* and *Neisseria sicca* (Mock 1) or the same combination but with *Streptococcus mutans* instead of N. sicca (Mock 2) were performed. We mixed the same amount of DNA from the 5 bacterial species to achieve a final concentration of 5 ng/µl (1 ng of each species). In order to estimate the proportion of each 16S rRNA gene in our mock community we proceed as in Johnson et al. [12]. Mass of DNA per genome was calculated based on the genome size of the strain multiplied by the mean weight of a base pair (1.079 × 10^–12^ ng/bp) and normalized by the copy number of the 16S rRNA gene. Two replicates of each mock were analyzed. Details of the bacterial strains, genome size and number of 16S rRNA gene copies are shown in Table 1.

**Table 1:** In-house mock communities’ components and genomic characteristics

**Mock 1**								
**DSM**	**Name**	**Refseq**	**16S rRNA gene copy number**	**Genome size (bp)**	**Mass of DNA per genome**^**1**^	**Nº genomes per ng**	**Nº 16S rRNA gene copies per ng**	**RA**^**2**^** (%)**
DSM 23056	***Actinomyces oris***	NZ_CP066060.1	3	3,184,721	3.436E-06	2.91 × 10^5^	8.73 × 10^5^	12.05
DSM 20482	***Fusobacterium nucleatum***	NZ_LN831027.1	5	2,443,126	2.636E-06	3.79 × 10^5^	1,9 × 10^6^	26.19
DSM 43762	***Rothia dentocariosa***	NC_014643.1	3	2,506,025	2.704E-06	3.7 × 10^5^	1.1 × 10^6^	15.32
DSM 20627	***Streptococcus oralis***	NZ_LR134336.1	4	1,931,548	2.084E-06	4.8 × 10^5^	1.9 × 10^6^	26.50
DSM 17713	***Neisseria sicca***	NZ_CP072524.1	4	2,566,407	2.769E-06	3.61 × 10^5^	1.44 × 10^6^	19.94
						Total	7.2 × 10^6^	100
**Mock 2**								
**DSM**	**Name**	**Refseq**	**16S rRNA gene copy number**	**Genome size (bp)**	**Mass of DNA per genome**^**1**^	**Nº genomes per ng**	**Nº 16S rRNA gene copies per ng**	**RA**^**2**^** (%)**
DSM 23056	***Actinomyces oris***	NZ_CP066060.1	3	3,184,721	3.436E-06	2.91 × 10^5^	8.73 × 10^5^	10.79
DSM 20482	***Fusobacterium nucleatum***	NZ_LN831027.1	5	2,443,126	2.636E-06	3.79 × 10^5^	1,9 × 10^6^	23.44
DSM 43762	***Rothia dentocariosa***	NC_014643.1	3	2,506,025	2.704E-06	3.7 × 10^5^	1.1 × 10^6^	13.71
DSM 20627	***Streptococcus oralis***	NZ_LR134336.1	4	1,931,548	2.084E-06	4.8 × 10^5^	1.9 × 10^6^	23.71
DSM 20627	***Streptococcus mutans***	NZ_LS483349.1	5	2,019,343	2.178E-06	4.6 × 10^5^	2.29 × 10^6^	28.35
						Total	8.09 × 10^6^	100

^1^Mass of DNA per genome. Number of genome bp * weigh of a bp (1.079 × 10–12 ng/bp)

^2^RA = Estimated relative abundance in percentage

### Illumina sequencing

The Illumina amplicon library preparation was performed following the 16S rRNA gene Metagenomic Sequencing Library Preparation Illumina protocol (Part #15,044,223 Rev. A). The primer used were 16S Amplicon Bakt_341F (TCGTCGGCAGCGTCAGATGTGTATAAGAGACA-GCCTACGGGNGGCWGCAG) and Bakt_805R (GTCTCGTGGGCTCGGAGATGTGTATAAGAGACAGG-ACTACHVGGGTATCTAATCC). These primers amplify the V3-V4 hypervariable regions of the gene, resulting in a single amplicon of about 460 bp. 25 cycles of PCR amplification were performed (denaturing at 95 ºC for 30 s, annealing at 55 ºC for 30 s and extension at 72 ºC for 30 s) and after cleanup an additional 8 cycles were performed to attach the dual indices and Illumina sequencing adapters. Negative controls were included. Following amplification, DNA was sequenced with an Illumina MiSeq Sequencer according to manufacturer’s instructions using the 2 × 300 bp paired-ends protocol.

### PacBio sequencing

The 27F (AGRGTTYGATYMTGGCTCAG) and 1492R (RGYTACCTTGTTACGACTT) universal primer set was used to amplify the full-length 16S rRNA gene from the genomic DNA. Both the forward and reverse 16S primers were tailed with sample specific PacBio barcode sequences to allow for multiplexed sequencing. Negative controls were included.

The KAPA HiFi Hot Start DNA Polymerase (KAPA Biosystems) was used to perform 27 cycles of PCR amplification, with denaturing at 95 °C for 30 s, annealing at 57 °C for 30 s and extension at 72 °C for 60 s. Post-amplification quality control was performed using the Fragment analyser (Agilent Technologies, Santa Clara, CA, USA). The DNA amplified from each sample was then pooled in equimolar concentration before starting the library preparation procedure. Amplified DNAs pool was processed with the SMRTbell Express Template Prep Kit 2.0 (PacBio, USA), following the Procedure & Checklist – Amplification of Full-Length 16S Gene with Barcoded Primers for Multiplexed SMRTbell® Library Preparation and Sequencing protocol (Part Number 101–599-700 Version 04, PACBIO). Fluorescence concentration and average size of the library pool obtained, were checked using Qubit HS DNA kit (Qubit Fluorometer, Invitrogen, USA) and Fragment Analyzer (Agilent Technologies, USA). Before loading the pool to the sequencer, sequencing primer annealing and polymerase binding steps were carried out, using the Sequel II Binding Kit 2.1 and the Sequel II DNA Internal Control Complex 1.0 (PacBio, USA). Sequencing was carried out using the Sequel II Sequencing Kit 2.0 (PacBio, USA) on the Sequel II PacBio system.

### Quality check and annotation

In order to process the Illumina sequences, we used the DADA2 R Statistics package (v1.20.0) [30, 31]. It first removed the primer sequences, then trimmed the reads by length and removed the reads exceeding 5 expected errors, dereplicated and estimated sequencing errors using loessErrfun. True sequence variants were inferred and the forward and reverse reads were merged together to obtain the full denoised sequences with a minimum overlapping region of 15 bp. ASVs identified as chimeric were removed and the remaining reads were annotated to SILVA v.138.1 database [32, 33].

Pacific Bioscience data were demultiplexed and CCS were called using the SMRT-Link analysis software (v9). A mean of 19 HiFi passes were used and the obtained CCS were submitted to the quality check pipeline following the DADA2 R Statistics package (v1.20.0) [30, 31] which, briefly, reorients the sequences, removes forward and reverse primers, filters and trims the sequences by length and average quality, dereplicates and estimates sequencing errors using PacBio error's model, and infers the compositions of the samples. ASVs identified as chimeric were removed and remaining ones were annotated using the naive Bayesian classifier method from DADA2 against the species train set of Silva138.1 [30, 32–34] database with a minimum bootstrap confidence of 80.

In addition, for both cases, ASVs with assigned genus but without exact matching at the species level were mapped against the same reference database but using Blastn with a minimum overlap of 100% and identity percentage of 97%. The best match is assigned only if the identity difference between the first and the second-best matches is more than 2% [35]. The taxonomic annotations were then used to compile contingency tables at every taxonomic rank.

### Platform comparison

The minimum number of reads in a sample annotated at the ASV level (4.5 × 10^3^) was used to perform rarefaction curves, richness, and diversity analyses at ASV and genus levels. To evaluate the similarities between sample types and platforms we executed a Principal Coordinates Analysis (PCoA) based on Bray–Curtis distance at the genus level as well as an analysis of variance using permutational multivariate analysis of variance using distance matrices (Adonis test) from the Vegan R package [34, 36]. Afterwards, the relative abundance was compared between sequencing technologies, using paired non-parametric Wilcoxon tests. Adjustment for multiple testing was performed through Benjamini–Hochberg method, considering significant values < 0.05 [37]. Spearman correlations between the relative abundance from each platform of the 20 most abundant genera were calculated. Statistical analysis were performed using R software [31].

## Results

### Sequencing performance

The sequencing outcome from the three types of human samples (gastrointestinal, subgingival plaque biofilm and saliva microbiomes), as well as from two custom-made mock communities obtained by sequencing the V3-V4 16S rRNA variable regions with Illumina Miseq and Full Length 16S rRNA gene with PacBio Sequel II sequencing are shown in Table 2.

**Table 2:** Number of reads sequenced and annotated by platform and sample type

**Platform**	**Sample Type**	**Nº Reads** Mean (SD)	**Filtered**^**1**^ Mean (SD)	**Reads length** mean	Total **ASV**^**2**^	Total Genera^2^	% reads assigned to **Genera**	% reads assigned to **Species**
Illumina	Mock 1	91,804 (16,260)	75,620 (13,350)	417.5 bp	7	6	100	58.33
	Mock 2	90,301 (856)	62,444 (44)	419.5 bp	15	5	100	69.27
	Saliva	94,079 (14,632)	70,661 (10,323)	418.1 bp	1414	138	96.46	52.79
	Subgingival	110,328 (44,092)	84,896 (36,315)	415.3 bp	1328	153	95.5	55.73
	Faeces	212,795 (122,662)	157,113 (94,087)	408.5 bp	1113	201	89.49	53.13
PacBio	Mock 1	26,065 (1431)	15,536 (870)	1455.5 bp	14	5	100	100
	Mock 2	33,620 (7213)	19,821 (2565)	1469 bp	17	4	99.99	99.73
	Saliva	21,151 (12,907)	17,444 (11,848)	1459.33 bp	2247	113	97.25	74.91
	Subgingival	13,982 (2163)	10,103 (2796)	1456.44 bp	1380	99	95.41	72.80
	Faeces	12,702 (2185)	7350 (2312)	1452.25 bp	796	97	89.72	61.93

^1^Filtered refers to number of sequences after quality check, primer trimmed, merged (only for Illumina dataset) and chimeric removal

^2^Total detected, excluding those with a number of reads < 10

Due to the library and run configuration, designed to have a similar number of samples per run with a sequencing cost within the same order of magnitude for both methodologies, we obtained a lower amount of reads in the case of PacBio technology for all types of samples (Table 2). Illumina sequences had a mean length of 414 bp after filtering and merge of the forward and revers reads, whereas PacBio sequences had a mean length of 1457 bp. On the other hand, Illumina sequences had an average quality score of 40, whereas PacBio consensus reads had a mean quality score of 90 (See Additional Fig. 1). However, the way that sequence quality is calculated in either methodology is intrinsically different due to the specific characteristics of final sequence assembly and the values are unfortunately not comparable.

After quality check and filtering we were able to annotate a mean of 12,500 reads per sample in PacBio whereas in Illumina the number of sequences was almost eightfold higher. The estimated number of ASV was larger in the PacBio platform for saliva and subgingival plaque biofilm samples, but lower in the faecal samples. Specifically, in the Illumina sequencing platform, 1414 ASVs were detected in saliva, 1328 in subgingival plaque biofilm and 1113 in faecal samples. In the case of PacBio sequencing, 2247 ASVs, 1380 ASVs and 796 ASVs were detected in saliva, subgingival plaque biofilm and faeces, respectively.

The degree of taxonomic assignment, especially at the species level, was higher in the PacBio sequencing platform. Specifically, during annotation, 94.79% of Illumina reads (saliva 96.46% ± 1.39; subgingival 95.50% ± 2.47; faeces 89.49% ± 7.41) and only 55.23% (saliva 52.79% ± 7.49; subgingival 55.73% ± 9.61; faeces 53.13% ± 12.68) reached the genus and species level, respectively. Meanwhile in PacBio, 95.06% (saliva 97.25% ± 1.86; subgingival 95.41% ± 3.28; faeces 89.72% ± 7.81) and 74.14% (saliva 74.91% ± 4.71; subgingival 72.8% ± 10.79; faeces 61.93% ± 19.1) were taxonomically assigned at the genus and species level, respectively.

### Assessment of taxonomic accuracy with mock communities

Two mock communities composed by five different species each were tested (Table 1). In both cases, Illumina sequences belonging to *Actinomyces* and part of those belonging to *Fusobacterium* and *Streptococcus* could not be assigned at the species level whereas the five bacteria in the mock community could be correctly identified with the PacBio sequencing platform. In addition, a false positive hit (corresponding to *Pediococcus parvulus*) was identified at low abundance (0.01%) with the Illumina platform in custom-made Mock 1 (See Additional Fig. 2).

Concerning the observed relative abundance in Mock 1, we observed an over representation of the genus *Neisseria* in both platforms (expected: 19.94%, Illumina: 25.84%, PacBio: 42.55%) to the detriment of *Actinomyces* (expected: 12.05%, Illumina: 5.47%, PacBio: 1.7%). In the case of Mock 2, *S. mutans* was overrepresented specially in PacBio platform (expected: 28.35%, I: 37.23%, P: 68.9%). This indicates that although the accuracy on the identification of the species was improved with PacBio platform, some species would be favored with this technology.

### Methodology comparison in complex human samples

When we compared the complex microbiome samples, rarefaction curves showed that sequencing depth was sufficient to cover bacterial diversity with both Illumina (I) or PacBio (P) sequencing in the three sample types (See Additional Fig. 3A). Regarding richness, the mean values of Chao1 index ranged from 130 to 360 at the ASV level depending on sample type (I-Saliva 341.77, P-Saliva 362.06, I-Subgingival 300.99, P-Subgingival 234.28, I-Faeces 259.1, P-Faeces 138.31), with only the faecal samples being significantly different between platforms (*p*-value < 0.001).

**Fig. 2:**
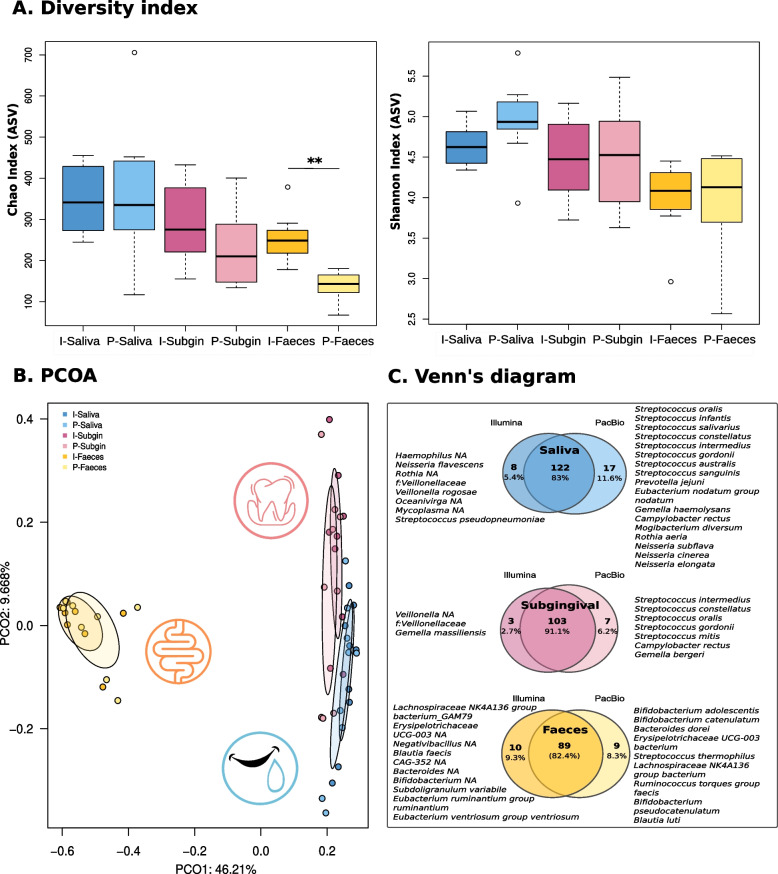
Bacterial richness, diversity and composition similarity between samples according to the sequencing platform. **A** On the left panel, boxplots show Chao index richness values; on the right, boxplots show Shannon index diversity values at the ASV level. **B** PCoA performed by Bray–Curtis distances on bacterial composition of all samples at the genus taxonomic level. **C** Venn’s diagram representing shared and unique species between platforms. Only species with a mean abundance > 0.1% were considered. * Stands for *p*-value < 0.05, ** for *p*-value < 0.01 in Wilcoxon tests. NA refers to bacterial genera that could not be assigned at the species level

This difference was not observed in Shannon diversity index, where mean values ranged between 4 and 5 (I-Saliva 4.66, P-Saliva 4.95, I-Subgingival 4.48, P-Subgingival 4.45, I-Faeces 3.99, P-Faeces 3.97) (Fig. 2A). However, at the genus level, the richness obtained in PacBio samples was significantly lower than in Illumina for subgingival biofilm (I-Subgingival 83.78, P-Subgingival 58.79, *p*-value 0.01) and faecal samples (I-Faeces 96.13, P-Faeces 47.19, *p*-value < 0.001) (See Additional Fig. 3C). In addition, at the genus level, faecal samples showed a significantly lower Shannon index (I-Faeces 3.12, P-Faeces 2.61, *p*-value 0.02) in PacBio compared to Illumina.

In order to assess the overall similarity in bacterial composition of each sample with their corresponding pair sequenced using a different approach, Bray Curtis distances, represented as PCoA at the genus level, were calculated (Fig. 2B). Samples clearly clustered by sample type (Adonis test: I-Saliva vs I-Subginival *p*-value 0.001, I-Saliva vs I-Faeces p-value 0.001, I-Subgingival vs I-Faeces p-value 0.002; P-Saliva vs P-Subgingival p-value 0.008, P-Saliva vs P-Faeces p-value 0.001, P-Subgingival vs P-Faeces *p*-value 0.002) and not by sequencing platforms (Adonis test: I-Saliva vs P-Saliva p-value 0.18, I-Subgingival vs P-Subgingival 0.38, I-Faeces vs P-Faeces *p*-value 0.89), showing that the two methods provided similar overall outcomes.

Regarding taxonomic composition, both platforms shared all the detected genera which represented more than 0.1% abundance. At the species level, more than 80% of them were shared between platforms (Fig. 2C), although 17 species in saliva, 7 in subgingival plaque biofilm and 9 in faeces were identified only with PacBio. As mentioned previously, the number of sequences annotated at the species level was higher in the case of PacBio. For example, in the case of the most abundant genus in saliva (*Streptococcus*), 93.4% of the sequences were unassigned at the species level in Illumina whereas with PacBio the unidentified reads only accounted for 29.7%. Similarly, the 32.5% of *Prevotella* sequences in subgingival plaque biofilm samples were unassigned at species level using Illumina whereas only the 13% of them could not be annotated using PacBio. Among the faecal samples, 99.97% of Illumina and 100% of PacBio sequences for *Faecalibacterium* were identified as *Faecalibacterium prausnitzii*. However, in the case of *Bifidobacterium*, 45.8% of the Illumina sequences were unassigned at the species level, whereas all the PacBio ones achieved species annotation (See Additional Fig. 4). Therefore, we observed an improvement in species assignment with PacBio technology in all three human samples.

**Fig. 3:**
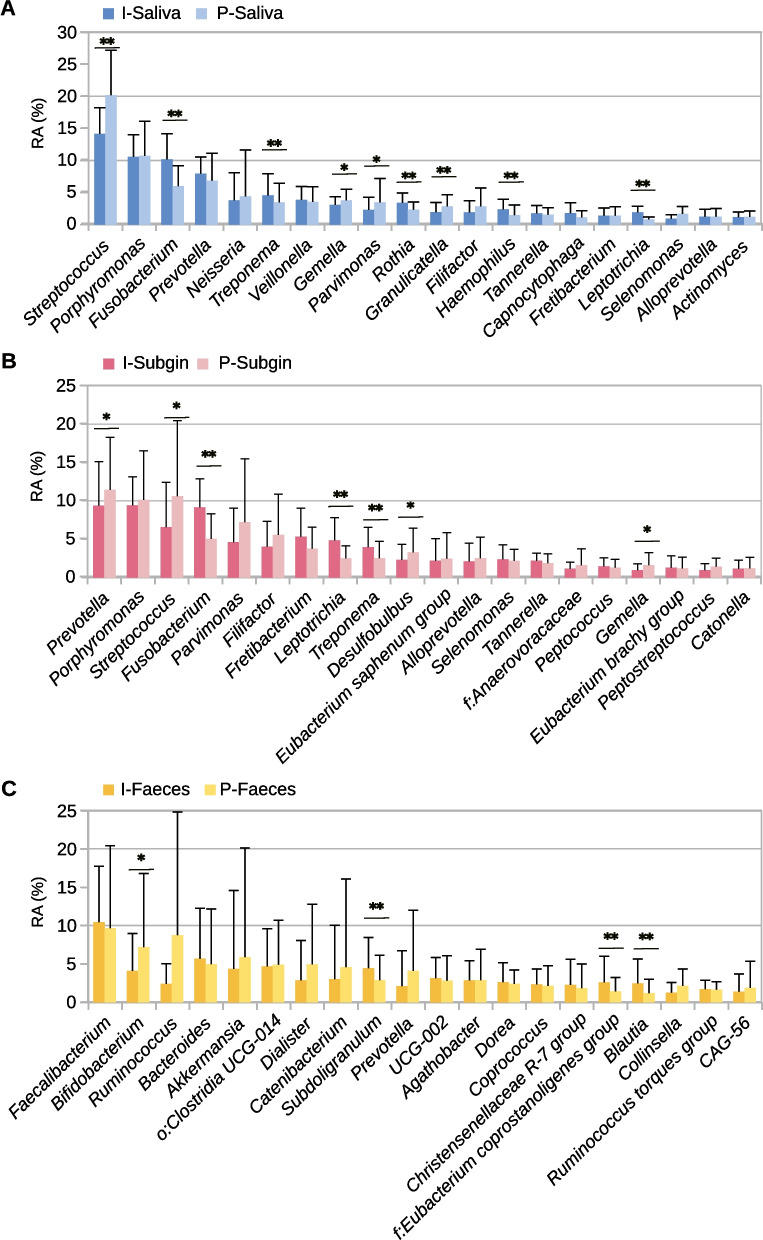
Differences in bacterial composition for human microbiome samples between Illumina (I-) and PacBio (P-) sequencing of the 16S rRNA gene. Mean relative abundance and standard deviation of the 20 most abundant genera of each sample type. *p-value < 0.05, **p-value < 0.01 in Wilcoxon test without correction for multiple tests

In saliva, the most abundant genera were *Streptococcus* (14.12% ± 4.07 I; 20.14% ± 7 P), *Porphyromonas* (10.54% ± 3.45 I; 10.72% ± 5.37 P), *Fusobacterium* (10.14% ± 3.98 I; 6.01% ± 3.16 P) and *Prevotella* (7.92% ± 2.6 I; 6.86% ± 4.25 P). Similarly, *Prevotella* (9.37% ± 5.76 I; 11.43% ± 6.85 P), *Porphyromonas* (9.42% ± 3.7 I; 10.14% ± 6.38 P), Streptococcus (6.59% ± 5.8 I; 10.63% ± 9.82 P) and *Fusobacterium* (9.15% ± 3.73 I; 5.04% ± 3.26 P) were found with high abundance in subgingival plaque biofilm samples. In the case of faecal samples, the most abundant genera were *Faecalibacterium* (10.46% ± 7.25 I; 9.68% ± 10.7 P), *Bifidobacterium* (4.11% ± 4.87 I; 7.2% ± 9.58 P), *Ruminococcus* (2.43% ± 2.60 I; 8.77% ± 15.98 P) and *Bacteroides* (5.69% ± 6.56 I; 4.98% ± 7.2 P) (Fig. 3).

Comparing the relative abundance of bacteria obtained, 34, 24, and 40 genera in saliva, subgingival plaque biofilm and faecal samples respectively, differed between platforms but none of these differences were significant when correcting for multiple testing (adjusted p-values). Notably, the *Streptococcus* genus had higher mean relative abundance (not significant) in PacBio than in Illumina platform in saliva and subgingival plaque biofilm samples whereas other relevant genera known to be oral pathogens like *Fusobacterium, Leptotrichia* and *Treponema* showed a trend for lower relative abundance (*p* < 0.05, adjusted *p*-value > 0.05) (Fig. 3). Taking all this into account, the results for complex microbiota composition in both technologies were quite similar with only slight variations in relative abundance. When Spearman correlations of the 20 most abundant genera between both platforms were calculated, 16, 18 and 17 out of 20 genera had a Spearman rho > 0.8 in saliva, subgingival plaque biofilm and faecal samples, respectively. In saliva, *Fusobacterium, Actinomyces, Selenomonas* and *Neisseria* had a correlation lower than 0.8 whereas in subgingival plaque biofilm only *Selenomonas* and *Treponema* were under this threshold. In the case of faecal samples, *Dorea, Collinsella* and *Ruminococcus* had the lowest Spearman rho values (See Additional Table 3).

## Discussion

The sequencing of full length 16S rRNA gene is a promising tool for analysing microbial composition because it improves the taxonomic annotation resolution power allowing to discern between closely related organisms at species or even clone level [12]. This has become a necessity since microbiome studies have proven that species from the same genus can have considerably different functional roles or be associated with either health or disease [11, 38]. Computer simulations show considerable improvement in taxonomic assignment errors rates with longer sequence length [12]. However, very few studies have compared the accuracy in taxonomic assignment when sequencing the whole (PacBio) or one or two variable regions (Illumina) of the 16S rRNA gene in real samples.

In the past, cloning of the full-length 16S rRNA gene and subsequent Sanger sequencing allowed accurate taxonomic assessment of environmental and human samples, although the low number of clones obtained – typically within a few hundred – only detected the most common organisms [39]. With the advent of 2^nd^ generation sequencing such as 454 pyrosequencing or Illumina sequencing, thousands of partial 16S rRNA gene sequences are easily achieved, but at the expense of less robust taxonomic assignment [40]. The arrival of 3^rd^ generation, single-molecule sequencing allows full-length 16S sequencing with high throughput capabilities, but their performance must be evaluated.

The aim of this study was therefore to compare sequencing performance and bacterial composition between Illumina and PacBio platforms to evaluate the reliability and eventual differences in human microbiome assessment. Illumina produced an eightfold higher throughput than PacBio at a lower cost, with an average sequence length of 414 bp vs 1456 bp after quality filtering, respectively. The lower sequencing output by PacBio, however, did not imply a lack of diversity assessment. For instance, all genera present at least at 0.1% relative proportion were detected by both methods, and at the species level, PacBio identified a higher number of annotated species than Illumina in all three sample types. The estimated richness, however, was higher in Illumina, although that extra number of ASVs corresponded to bacteria below 0.1% that could not be annotated at the species level. Thus, although a higher cost would be needed for PacBio sequencing to achieve an equivalent number of sequences per sample, our data suggest that this is not necessary to represent the main diversity of a sample.

As expected, a higher proportion of reads were annotated at the species level in the case of PacBio platform (74% of the reads, vs 55% in Illumina). Even though PacBio successfully annotated more reads at the species level, 25% of the sequences could still not be unequivocally assigned. A possible reason for the lack of full assignment is that part of this 25% corresponds to under-represented species in databases, including the high number of uncultured bacteria that are obtained by metagenomic-assembled genomes (MAGs), as suggested by Pasolli et al., [41]. As other authors have already discussed, the selection of databases could have an important impact on the description of microbiome profiles. After about 15 years in which 2^nd^ generation sequencing technologies were dominating the microbiome profiling analysis, the databases are consequently not updated or, even worst, not prepared to assume the annotation required by the usage of full length 16S rRNA query [42]. Therefore, working in databases curation and update is still needed to improve the annotation power for 3^rd^ generation sequencing output.

Host-associated microbiome studies usually compare the diversity found in samples (e.g., health vs disease conditions) as an indication of alterations in the microbiome. Given that taxa can be more precisely differentiated at the species level using PacBio, one would expect this sequencing platform would show higher diversity than Illumina. Although the throughput obtained with Illumina sequencing was higher than with PacBio, the diversity indexes were similar between platforms, with the exception of faecal samples. Is probably a consequence of this samples having the lowest throughput in the PacBio platform with less than 10,000 reads per sample. So even if it does not appear to be necessary to reach a sequencing depth as high as with Illumina, too few sequences could prevent the detection of low abundant taxa. Other potential reasons include a higher false variants rate in this platform, which would inflate richness values. This hypothesis is supported by the results obtained when comparing the custom-made mock communities between platforms, where several unclassified species were detected in Illumina platform, and none was seen in PacBio. The errors introduced by Illumina could result in the emergence of these unclassified species, increasing diversity. Other authors already pointed out this increased noise in Illumina when mock communities were assessed [43]. This must be taken into account in future studies when diversity in samples sequenced by 2nd and 3rd generation sequencing technologies are compared.

In general, we observed a high consistency between bacterial composition obtained with both platforms for the three types of samples, at least up to the genus level. The comparison of the presence/absence of taxonomically assigned bacteria showed that most genera were present in both platforms. This suggests that outputs from both sequencing platforms would be generally suitable for future comparisons at the genus level. Similar results were reported by Matsuo Y, et al. who evaluated the performance of FL-16S rRNA gene sequencing by MinION™ technology in human faecal samples [44]. This indicates that the technical improvements in the quality and in the processing pipelines of the 3rd generation technologies currently allow to have reliable results, opening the door to make a definitive move towards complete 16 rRNA gene sequencing.

However, our results suggest that the abundance of bacteria of high relevance for oral microbiome studies such as *Streptococcus* or *Fusobacterium* and for intestinal microbiome studies such as *Bifidobacterium* tend to be different between platforms. This lack of congruency for several bacteria was confirmed by correlation analysis between bacterial composition in the two platforms, with some bacterial genera showing correlation coefficients below 0.8. In addition, an over-representation of *Neisseria* and under-representation of *Actinomyces* was observed in the mock community. These variations could be due to methodological differences [45] such as specificity of primers (giving rise to preferential amplification of some taxa), stringency of PCR conditions (widening the number of species amplified), read length (preventing/allowing the sequencing of high-resolution regions for specific bacteria) or coverage. It must be taken into consideration that the primers for the V3-V4 hypervariable region were originally designed based on environmental samples [46] although they are used for niches as different as soil or the human oral cavity. However, primers used in 3^rd^ generation sequencing platforms are designed on a wider number of species in public databases, potentially conferring less amplification bias. Thus, future host-associated microbiome studies using PacBio technology should take into account these differential features when comparing with previous studies using Illumina technology.

The results presented in the current manuscript suggest that samples sequenced using Illumina and PacBio are mostly comparable but future studies should take into account that some important genera can be differently represented in relative abundance depending on whether a partial region or the whole gene is sequenced. Considering that PacBio reads were assigned at the species level with higher rates than Illumina, our data support the use of PacBio technology for future microbiome studies with higher resolution. Given that 16S rRNA sequencing in both platforms relies on several PCR steps that could introduce different biases, experimental and computational procedures that allow the mitigation of the bias [47, 48] should also be implemented.

### Supplementary Information

**Additional file 1.** Supplementary figures.

**Additional file 2.** Supplementary tables.

## Data Availability

The raw sequencing data during the current study are available in the SRA repository with the accession number PRJNA933120. [https://www.ncbi.nlm.nih.gov/bioproject/PRJNA933120].

## Abbreviations

PacBio: Pacific Bioscience
SMRT: Single Molecule Real-Time
CCS: Circular consensus sequencing
FL-16S: rRNA gene Full length 16S rRNA gene
PBS: Phosphate-buffered saline
ASV: Amplicon sequence variants
PCoA: Principal Coordinates Analysis

## References

[1] Woese CR, Fox GE (1977). Phylogenetic structure of the prokaryotic domain: The primary kingdoms. Proc Natl Acad Sci U S A.

[2] Starke R, Pylro VS, Morais DK (2021). 16S rRNA Gene Copy Number Normalization Does Not Provide More Reliable Conclusions in Metataxonomic Surveys. Microb Ecol.

[3] Stackebrandt E, Goebel BM (1994). Taxonomic note: A place for DNA-DNA reassociation and 16S rRNA sequence analysis in the present species definition in bacteriology. Int J Syst Bacteriol.

[4] Simon-Soro A, Tomas I, Cabrera-Rubio R, Catalan MD, Nyvad B, Mira A (2013). Microbial Geography of the Oral Cavity. J Dent Res.

[5] Lazarevic V, Whiteson K, Huse S, Hernandez D, Farinelli L, Osterås M (2009). Metagenomic study of the oral microbiota by Illumina high-throughput sequencing. J Microbiol Methods.

[6] Klindworth A, Pruesse E, Schweer T, Peplies J, Quast C, Horn M (2013). Evaluation of general 16S ribosomal RNA gene PCR primers for classical and next-generation sequencing-based diversity studies. Nucleic Acids Res.

[7] Polz MF, Cavanaugh CM (1998). Bias in Template-to-Product Ratios in Multitemplate PCR. Appl Environ Microbiol.

[8] Pereira-Marques J, Hout A, Ferreira RM, Weber M, Pinto-Ribeiro I, Van Doorn LJ (2019). Impact of host DNA and sequencing depth on the taxonomic resolution of whole metagenome sequencing for microbiome analysis. Front Microbiol..

[9] Claesson MJ, Wang Q, O’Sullivan O, Greene-Diniz R, Cole JR, Ross RP (2010). Comparison of two next-generation sequencing technologies for resolving highly complex microbiota composition using tandem variable 16S rRNA gene regions. Nucleic Acids Res.

[10] Dzidic M, Collado MC, Abrahamsson T, Artacho A, Stensson M, Jenmalm MC (2018). Oral microbiome development during childhood: an ecological succession influenced by postnatal factors and associated with tooth decay. ISME J.

[11] Giacomini JJ, Torres-Morales J, Dewhirst FE, Borisy GG, Mark Welch JL (2023). Site Specialization of Human Oral Veillonella Species. Microbiol Spectr.

[12] Johnson JS, Spakowicz DJ, Hong B-Y, Petersen LM, Demkowicz P, Chen L (2019). Evaluation of 16S rRNA gene sequencing for species and strain-level microbiome analysis. Nat Commun.

[13] Leggett RM, Clark MD (2017). A world of opportunities with nanopore sequencing. J Exp Bot.

[14] Benítez-Páez A, Sanz Y (2017). Multi-locus and long amplicon sequencing approach to study microbial diversity at species level using the MinION™ portable nanopore sequencer. Gigascience.

[15] Eid J, Fehr A, Gray J, Luong K, Lyle J, Otto G (2009). Real-time DNA sequencing from single polymerase molecules. Science.

[16] LaPierre N, Egan R, Wang W, Wang Z (2019). De novo Nanopore read quality improvement using deep learning. BMC Bioinformatics.

[17] Wenger AM, Peluso P, Rowell WJ, Chang PC, Hall RJ, Concepcion GT (2019). Accurate circular consensus long-read sequencing improves variant detection and assembly of a human genome. Nat Biotechnol.

[18] Callahan BJ, Wong J, Heiner C, Oh S, Theriot CM, Gulati AS (2019). High-throughput amplicon sequencing of the full-length 16S rRNA gene with single-nucleotide resolution. Nucleic Acids Res.

[19] Eriksson L, Lif Holgerson P, Johansson I (2017). Saliva and tooth biofilm bacterial microbiota in adolescents in a low caries community. Sci Rep.

[20] Wang Y, Zhang J, Chen X, Jiang W, Wang S, Xu L (2017). Profiling of Oral Microbiota in Early Childhood Caries Using Single-Molecule Real-Time Sequencing. Front Microbiol.

[21] He Q, Kwok LY, Xi X, Zhong Z, Ma T, Xu H, et al. The meconium microbiota shares more features with the amniotic fluid microbiota than the maternal fecal and vaginal microbiota. Gut Microbes. 2020;12(1):1794266. 10.1080/19490976.2020.1794266. 10.1080/19490976.2020.1794266 PMC7524391 32744162

[22] Ihara Y, Takeshita T, Kageyama S, Matsumi R, Asakawa M, Shibata Y, et al. Identification of Initial Colonizing Bacteria in Dental Plaques from Young Adults Using Full-Length 16S rRNA Gene Sequencing. mSystems. 2019;4(5):e00360-19. 10.1128/mSystems.00360-19. 10.1128/mSystems.00360-19 PMC6722423 31481603

[23] Yang X, He L, Yan S, Chen X, Que G (2021). The impact of caries status on supragingival plaque and salivary microbiome in children with mixed dentition: a cross-sectional survey. BMC Oral Health.

[24] Wu Y-F, Lee W-F, Salamanca E, Yao W-L, Su J-N, Wang S-Y (2021). Oral Microbiota Changes in Elderly Patients, an Indicator of Alzheimer’s Disease. International Journal of Environmental Research and Public Health Article.

[25] Rayamajhi N, Cheng CHC, Catchen JM. Evaluating Illumina-, Nanopore-, and PacBio-based genome assembly strategies with the bald notothen, Trematomus borchgrevinki. G3: Genes|Genomes|Genetics. 2022;12(11):jkac192. 10.1093/g3journal/jkac192. 10.1093/g3journal/jkac192 PMC9635638 35904764

[26] Cook R, Brown N, Rihtman B, Michniewski S, Redgwell T, Clokie M, et al. The long and short of it: Benchmarking viromics using Illumina, Nanopore and PacBio sequencing technologies. bioRxiv. 10.1101/2023.02.12.527533. 10.1099/mgen.0.001198 PMC10926689 38376377

[27] Zhang J, Su L, Wang Y, Deng S. Improved High-Throughput Sequencing of the Human Oral Microbiome: From Illumina to PacBio. Can J Infect Dis Med Microbiol. 2020;2020:6678872. 10.1155/2020/6678872. 10.1155/2020/6678872 PMC7748900 33381248

[28] Weisburg WG, Barns SM, Pelletier DA, Lane DJ (1991). 16S ribosomal DNA amplification for phylogenetic study. J Bacteriol.

[29] Tonetti MS, Greenwell H, Kornman KS (2018). Staging and grading of periodontitis: Framework and proposal of a new classification and case definition. J Periodontol.

[30] Callahan BJ, McMurdie PJ, Rosen MJ, Han AW, Johnson AJA, Holmes SP (2016). DADA2: High-resolution sample inference from Illumina amplicon data. Nat Methods.

[31] R Core Team. A language and environment for statistical computing. R Foundation for Statistical Computing. 2012;10:11–8.

[32] Quast C, Pruesse E, Yilmaz P, Gerken J, Schweer T, Yarza P (2013). The SILVA ribosomal RNA gene database project: Improved data processing and web-based tools. Nucleic Acids Res.

[33] Yilmaz P, Parfrey LW, Yarza P, Gerken J, Pruesse E, Quast C (2014). The SILVA and “all-species Living Tree Project (LTP)” taxonomic frameworks. Nucleic Acids Res.

[34] McLaren MR, Callahan BJ. Silva 138.1 prokaryotic SSU taxonomic training data formatted for DADA2. 2021. 10.5281/ZENODO.4587955.

[35] Rosier BT, Palazón C, García-Esteban S, Artacho A, Galiana A, Mira A (2021). A Single Dose of Nitrate Increases Resilience Against Acidification Derived From Sugar Fermentation by the Oral Microbiome. Front Cell Infect Microbiol.

[36] Oksanen J, Simpson GL, Blanchet FG, Kindt R, Legendre P, Minchin PR, et al. Vegan: Community Ecology Package; 2022. https://CRAN.R-project.org/package=vegan.

[37] Benjamini Y, Hochberg Y (1995). Controlling the false discovery rate: A practical and powerful approach to multiple testing. J R Stat Soc.

[38] Utter DR, Borisy GG, Eren AM, Cavanaugh CM, Mark Welch JL (2020). Metapangenomics of the oral microbiome provides insights into habitat adaptation and cultivar diversity. Genome Biol.

[39] Pace NR, Stahl DA, Lane DJ, Olsen GJ. The Analysis of Natural Microbial Populations by Ribosomal RNA Sequences. 1986. p. 1–55. In: Marshall KC, editor. Advances in Microbial Ecology. Advances in Microbial Ecology, vol 9. Boston: Springer; 10.1007/978-1-4757-0611-6_1.

[40] Degnan PH, Ochman H (2012). Illumina-based analysis of microbial community diversity. ISME J.

[41] Pasolli E, Asnicar F, Manara S, Zolfo M, Karcher N, Armanini F (2019). Extensive Unexplored Human Microbiome Diversity Revealed by Over 150,000 Genomes from Metagenomes Spanning Age, Geography, and Lifestyle. Cell.

[42] Sierra MA, Li Q, Pushalkar S, Paul B, Sandoval TA, Kamer AR (2020). The Influences of Bioinformatics Tools and Reference Databases in Analyzing the Human Oral Microbial Community. Genes..

[43] Szoboszlay M, Schramm L, Pinzauti D, Scerri J, Sandionigi A, Biazzo M. Nanopore Is Preferable over Illumina for 16S Amplicon Sequencing of the Gut Microbiota When Species-Level Taxonomic Classification, Accurate Estimation of Richness, or Focus on Rare Taxa Is Required. Microorganisms. 2023;11(3):804. 10.3390/microorganisms11030804. 10.3390/microorganisms11030804 PMC10059749 36985377

[44] Matsuo Y, Komiya S, Yasumizu Y, Yasuoka Y, Mizushima K, Takagi T (2021). Full-length 16S rRNA gene amplicon analysis of human gut microbiota using MinION™ nanopore sequencing confers species-level resolution. BMC Microbiol.

[45] D’Amore R, Ijaz UZ, Schirmer M, Kenny JG, Gregory R, Darby AC (2016). A comprehensive benchmarking study of protocols and sequencing platforms for 16S rRNA community profiling. BMC Genomics.

[46] Herlemann DP, Labrenz M, Jürgens K, Bertilsson S, Waniek JJ, Andersson AF (2011). Transitions in bacterial communities along the 2000 km salinity gradient of the Baltic Sea. ISME J.

[47] Silverman JD, Bloom RJ, Jiang S, Durand HK, Dallow E, Mukherjee S (2021). Measuring and mitigating PCR bias in microbiota datasets. PLoS Comput Biol.

[48] Sipos R, Székely AJ, Palatinszky M, Révész S, Márialigeti K, Nikolausz M (2007). Effect of primer mismatch, annealing temperature and PCR cycle number on 16S rRNA gene-targetting bacterial community analysis. FEMS Microbiol Ecol.

