# Supplementary material for: Full-length 16S rRNA gene sequencing by PacBio improves taxonomic resolution in human microbiome samples
Source: BMC Genomics. 2024 Mar 25;25:310. doi: 10.1186/s12864-024-10213-5 (PMC10964587; doi:10.1186/s12864-024-10213-5)
Supplement: Supplementary file 1 — Additional file 1. Supplementary figures. [file 12864_2024_10213_MOESM1_ESM.pdf]

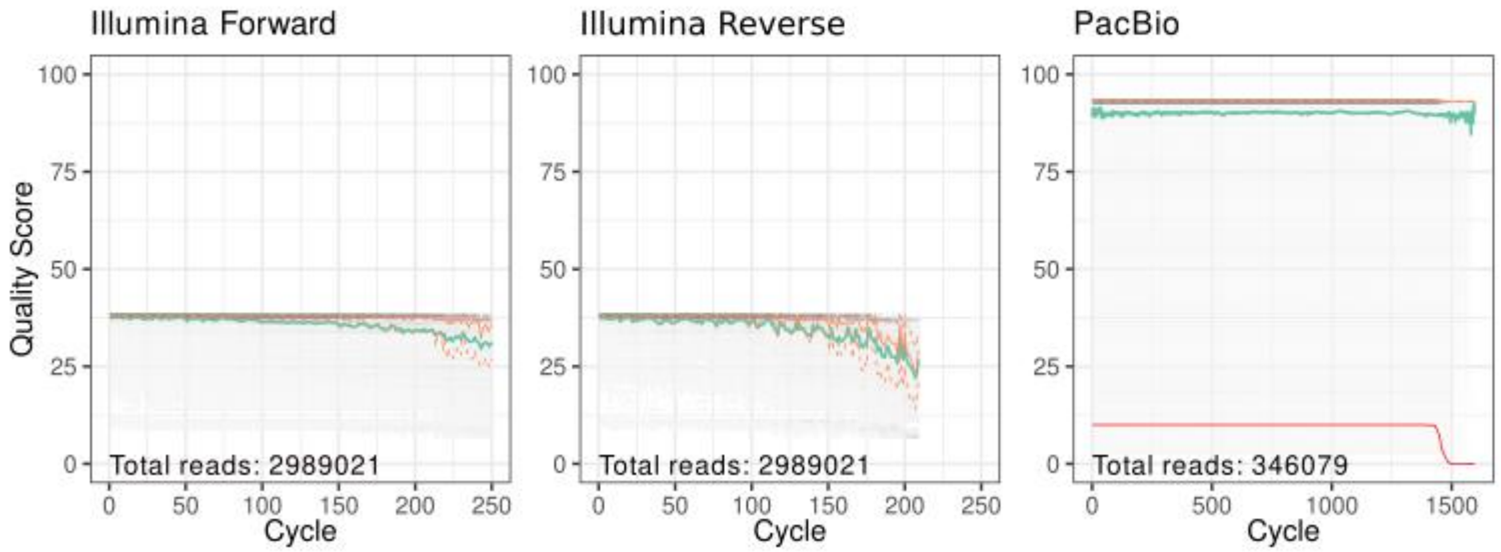

Additional Fig 1. Quality scores of sequences after filtering obtained by Illumina and PacBio platform. All human samples (n=26) were aggregated for visualization. Quality score at each base position was represented as a heat map in gray scale. Green line shows the mean and orange lines represent the quartiles values. Red line shows the scaled proportion of reads that extend to at least that position. In the case of PacBio platform consensus sequence was represented, therefore, the values are not comparable.

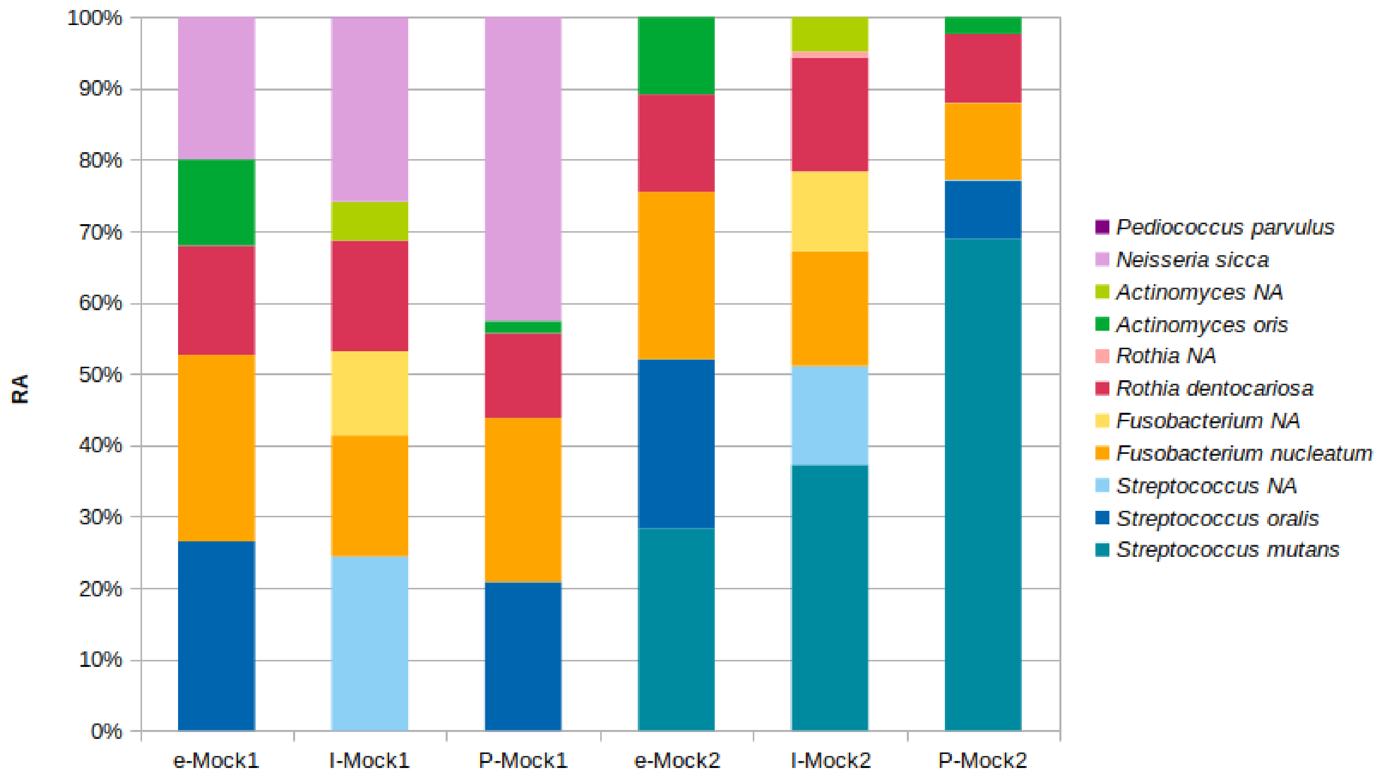

Additional Fig 2. Costume mock communities. Relative abundance at species level of costume mock communities. e-Mock, stands for theoretical calculated composition of the custom made mock communities.(I-: Illumina platform, P-: PacBio platform)

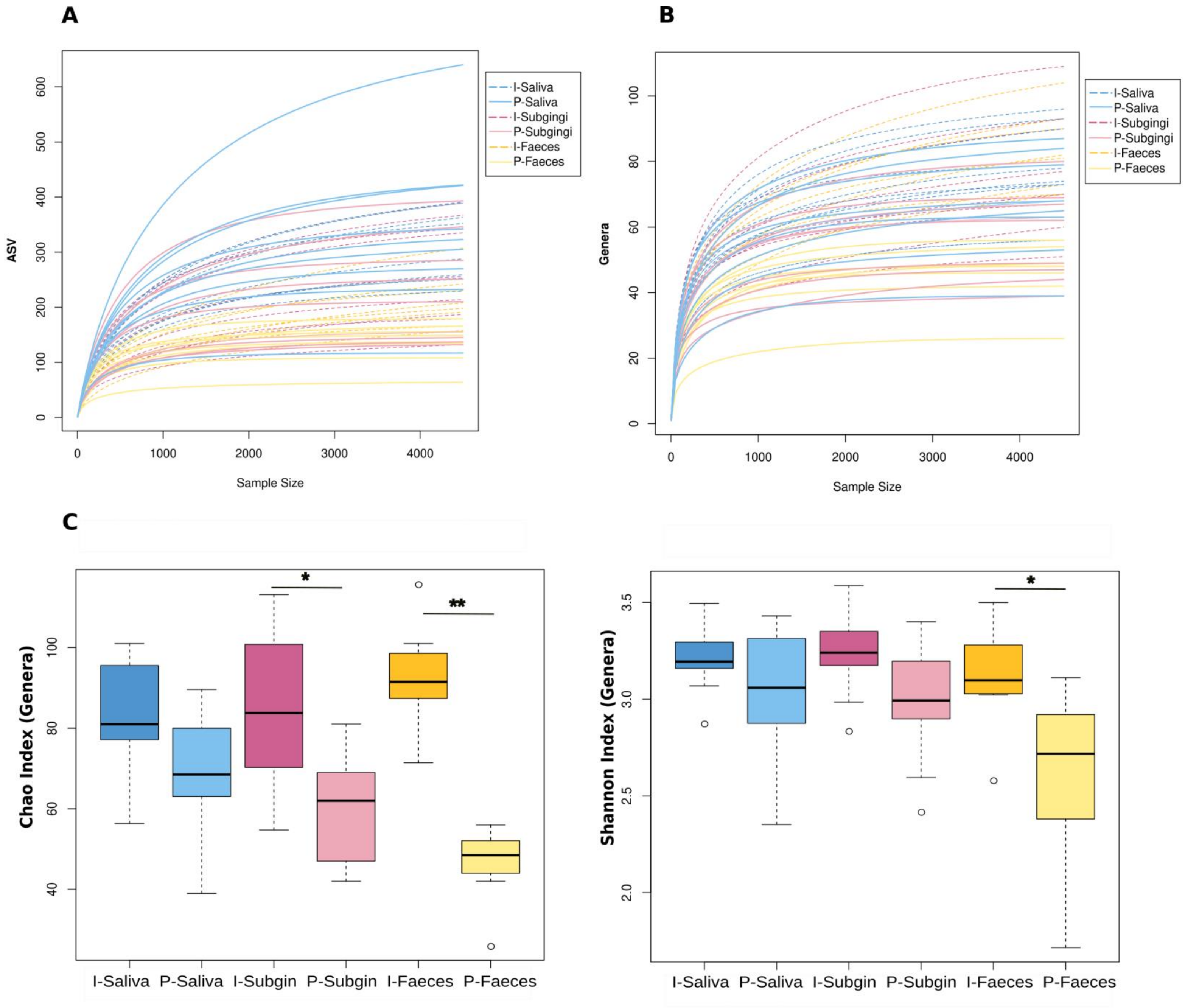

Additional Fig 3. **A-B**. Rarefaction curves at ASV (left) and genus (right) levels. Solid lines and light colours represent samples sequenced by PacBio while dashed and dark lines represent Illumina sequences. **C** Boxplots represent Chao index richness values (left), and Shannon index diversity values (right) at the genus taxonomic level. * p-value <0.05, ** p-value <0.01 in Wilcoxon tests.

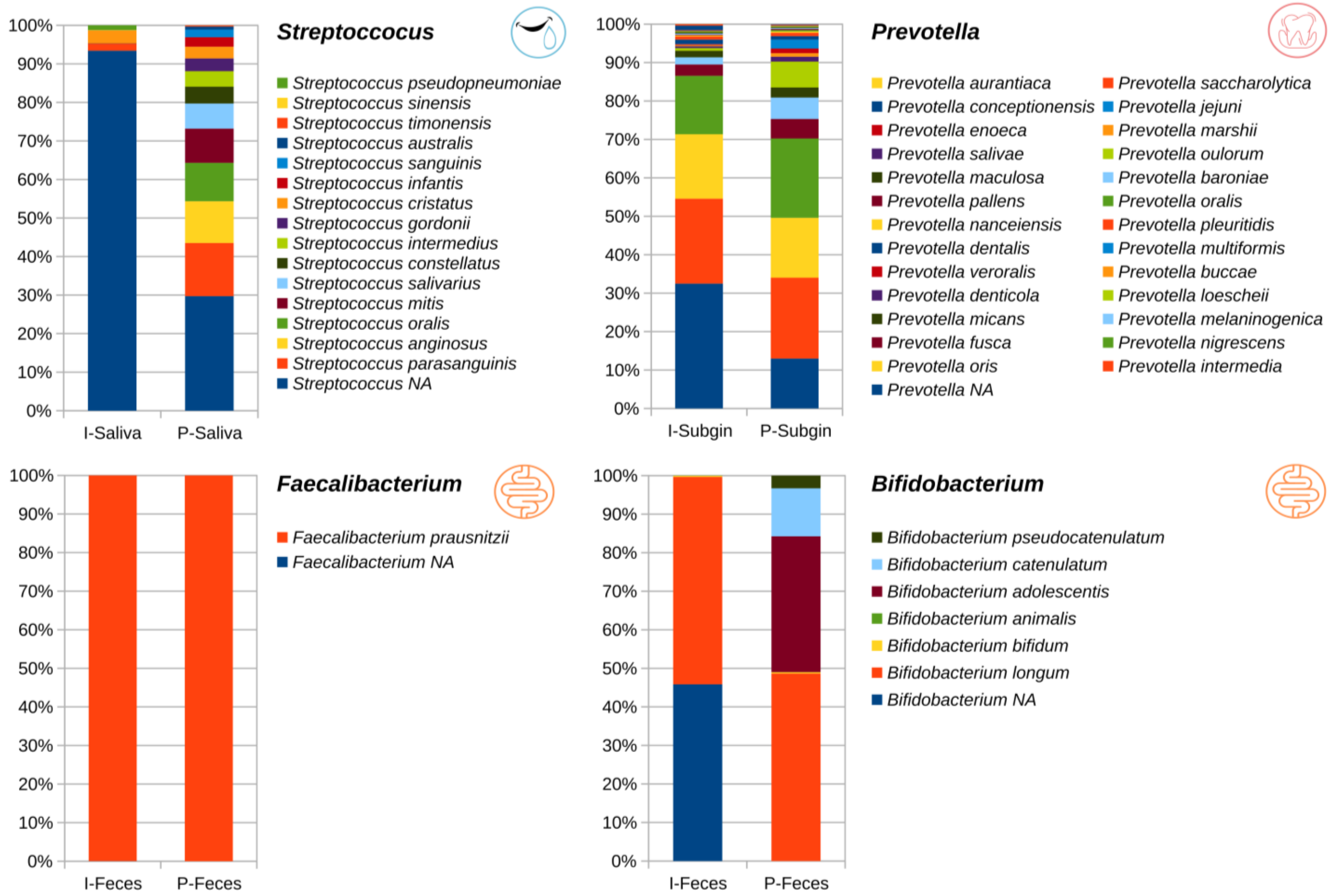

Additional Fig 4. Species level assignment. Assignment at the species level of the most abundant genera in each type of sample. Percentage of reads which represents each species among the total reads assigned to the respective genera on each platform.
